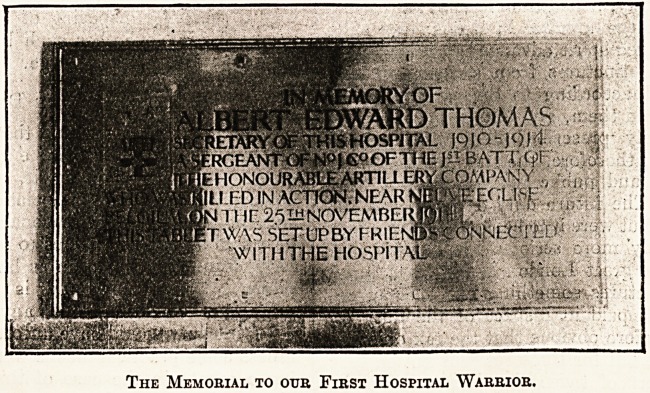# Albert Thomas, Our First Hospital Warrior

**Published:** 1915-05-08

**Authors:** 


### ALBERT THOMAS, OUR FIRST HOSPITAL WARRIOR.

### His Memorial, his Work and his Friends.

In The Hospital of December 5, 1914, p. 211, we published an account of the circumstances attending the death of Sergeant A. E. Thomas, who was the first hospital secretary to be killed in action in the Great War. In this connection we have to record an incident of more than usual interest at the annual general meeting of the Hampstead General and North-West London Hospital, which was held at the hospital on Tuesday, April 27 last. At the close of the meeting the Chairman, the Mayor of Hampstead (Mr. E. A. O'Bryen), asked the President of the hospital, His Imperial Highness the Grand Duke Michael of Russia, to unveil a memorial tablet that had been presented by friends of the late secretary, which the Council had placed in the main corridor of the hospital. Before the unveiling His Imperial Highness referred in eulogistic and sympathetic terms to the life and career of Mr. A. E. Thomas, who had been secretary to the hospital from July 1910. He had been called up for service with his regiment, the H.A.C., early in the commencement of the war, and proved himself an active and valiant soldier. Mr. Thomas died as he had lived—at the post of duty—full of zeal and devotion to his work, like the brave man he was. Indeed, the circumstances and manner of his death must almost be a consolation to his mother and family. The President dwelt with praise and gratitude on the splendid work Mr. Thomas did as secretary to the hospital. H.I.H. declared, indeed, that he had always felt through Mr. Thomas's instrumentality that the President was most intimately connected with the institution, in the management of which he was kept so well informed that everything that happened was brought to his notice. Mr. Thomas served his hospital well and faithfully, with great and lasting advantage to the institution and all associated with him in its work.

The Memorial to our First Hospital Warrior.

At the conclusion of his eloquent speech His Imperial Highness the Grand Duke Michael of Russia unveiled a handsome brass-framed tablet bearing an inscription and the illuminated arms of the Honourable Artillery Company, in which regiment Mr. Thomas was a sergeant. It is recorded that Mr. Thomas's last words were "Stick to it, boys; give it 'em."

The tablet was subscribed for by friends connected with the hospital.

At the close of the meeting a representative of the subscribers to the tablet handed a handsome travelling clock in a case to Mrs. Thomas, the mother, which bore the inscription, "In Memory of A. E. Thomas, Secretary of the Hampstead General Hospital, 1910-1914."

## Figures and Tables

**Figure f1:**